# A wearable chemical–electrophysiological hybrid biosensing system for real-time health and fitness monitoring

**DOI:** 10.1038/ncomms11650

**Published:** 2016-05-23

**Authors:** Somayeh Imani, Amay J. Bandodkar, A. M. Vinu Mohan, Rajan Kumar, Shengfei Yu, Joseph Wang, Patrick P. Mercier

**Affiliations:** 1 Department of Electrical and Computer Engineering, University of California, San Diego, La Jolla, California 92093-0407, USA; 2 Department of Nanoengineering, University of California, San Diego, La Jolla, California 92093-0448, USA

## Abstract

Flexible, wearable sensing devices can yield important information about the underlying physiology of a human subject for applications in real-time health and fitness monitoring. Despite significant progress in the fabrication of flexible biosensors that naturally comply with the epidermis, most designs measure only a small number of physical or electrophysiological parameters, and neglect the rich chemical information available from biomarkers. Here, we introduce a skin-worn wearable hybrid sensing system that offers simultaneous real-time monitoring of a biochemical (lactate) and an electrophysiological signal (electrocardiogram), for more comprehensive fitness monitoring than from physical or electrophysiological sensors alone. The two sensing modalities, comprising a three-electrode amperometric lactate biosensor and a bipolar electrocardiogram sensor, are co-fabricated on a flexible substrate and mounted on the skin. Human experiments reveal that physiochemistry and electrophysiology can be measured simultaneously with negligible cross-talk, enabling a new class of hybrid sensing devices.

## Abstract (web summary)

Wearable sensors can provide continuous, convenient feedback for users but typically focus on a small number of physiological parameters. Here, the authors report a skin-worn sensing system that combines a biosensor for lactate detection with an electrocardiogram in one patch, with applications for exercise monitoring.

## Graphical abstract

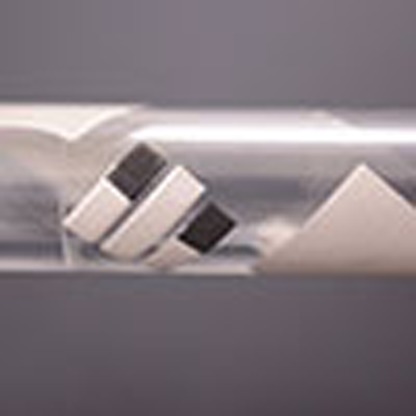

Wearable sensors present an exciting opportunity to measure human physiology in a continuous, real-time and non-invasive manner^1^,^2^. Recent advances in hybrid fabrication techniques have enabled the design of wearable sensing devices in thin, conformal form factors that naturally comply with the smooth curvilinear geometry of human skin, thereby enabling intimate contact necessary for robust physiological measurements^1^,^3^,^4^. Development of such epidermal electronic sensors has enabled devices that can monitor respiration rate^5^,^6^,^7^, heart rate^8^,^9^, electrocardiograms^4^,^10^,^11^,^12^, blood oxygenation^13^, skin temperature^14^,^15^, bodily motion^16^,^17^,^18^,^19^,^20^, brain activity^21^,^22^,^23^ and blood pressure^24^,^25^. To date, most systems have targeted only a single measurement at a time, and most such sensors measure only physical and electrophysiological parameters, significantly limiting monitoring and diagnostic opportunities. For example, the human body undergoes complex physiological changes during physical activities such as exercise^26^,^27^, and monitoring the physiologic effect of physical activity can be important for a wide variety of subjects ranging from athletes to the elderly^28^,^29^,^30^. However, current wearable devices that only measure heart rate, motion and electrocardiogram provide an incomplete picture of the complex physiological changes taking place. As a result, further progress in the area of wearable sensors must include new, relevant sensing modalities, and must integrate these different modalities into a single platform for continuous, simultaneous sensing of multiple parameters relevant to a wide range of conditions, diseases, health and performance states.

Inclusion of chemical measurements can provide extremely useful insights not available from physical or electrophysiological sensors^31^. Chemical information can be conventionally acquired via clinical labs or point-of-care devices^32^,^33^,^34^; unfortunately, such approaches do not support continuous, real-time measurements, therefore limiting their utility to applications where stationary, infrequent tests are sufficient. While recent work, including our own, has demonstrated that chemicals such as electrolytes and metabolites can be measured continuously using epidermal electronics on the skin^35^,^36^,^37^,^38^, or through non-invasive monitoring of other body fluids^38^,^39^,^40^, these devices measure only a single parameter at once, and are not integrated with other sensing modalities. Recently, Gao *et al*.^41^ demonstrated a wearable patch that can simultaneously track levels of metabolites and electrolytes in human sweat. However, electrophysiology sensors were not included, and such multimodal sensor fusion is crucial to obtain a more comprehensive knowledge about a wearer's well-being.

Here, we introduce a wearable device that can simultaneously measure chemical and electrophysiological parameters in the form factor of a single epidermal patch. The hybrid wearable, termed here as a Chem–Phys patch, comprises a screen-printed three-electrode amperometric lactate biosensor and two electrocardiogram electrodes, enabling concurrent real-time measurements of lactate and electrocardiogram. When used in physical-exertion monitoring, electrocardiogram measurements can help monitor heart health and function, while sweat lactate can be used to track an individual's performance and exertion level, and is also an important biomarker for tissue oxygenation and pressure ischaemia^42^,^43^,^44^,^45^,^46^,^47^. Although prior work has demonstrated separate wearable electrocardiogram and lactate sensors, these devices were fabricated on separate platforms and thus mandate applying multiple patches on the human body, which is inconvenient and can deter long-term use. By combining a lactate biosensor and an electrocardiogram sensor, the new Chem–Phys hybrid wearable patch represents a powerful platform capable of simultaneously tracking both physicochemical and electrophysiological attributes, thus providing a more comprehensive view of a person's health status than current wearable fitness monitors.

The Chem–Phys hybrid patch was fabricated by leveraging screen-printing technology on a thin, highly flexible polyester sheet that conforms well with the complex three-dimensional (3D) morphology of human skin to provide a low-noise signal. The working electrode of the lactate biosensor was functionalized and coated with a biocompatible biocatalytic layer (lactate oxidase (LOx)-modified prussian blue). The three amperometric electrodes were separated from the Ag/AgCl electrocardiogram electrodes via a printed hydrophobic layer to maximize sensor stability and signal-to-noise ratio even in the presence of significant perspiration. The dimensions of the electrodes and the inter-electrode distances have been optimized based on the human trials to acquire a clean electrocardiogram signal and lactate response with minimal interference between the two sensors. The two sensors were interfaced to a custom-printed circuit board (PCB) featuring a potentiostat, an electrocardiogram analogue front-end (AFE), and a bluetooth low-energy (BLE) radio for wireless telemetry of the results to a mobile platform, such as a smartphone or laptop. The hybrid sensing system was tested on three human subjects during exercise on a stationary bicycle, showing that lactate and electrocardiogram can be measured simultaneously with negligible co-interference. Electrocardiogram data was found to be similar to the data collected from standard electrode types, and extracted heart rate correlated well to commercial heart rate detectors. A control experiment, where an enzyme-free amperometric sensor was applied to a perspiring human subject, corroborated the lactate sensor's sensitivity and selectivity towards on-body detection of physiologic lactate levels. The promising data obtained in this work thus supports the possibility of developing more advanced hybrid wearable sensors that involve complex integration of several physical and chemical sensors on the same platform for monitoring many relevant modalities.

## Results

### Hybrid patch design rationale and fabrication

The Chem–Phys hybrid multi-sensor system must be compact and easy to wear in a location that offers adequate access to both electrocardiogram signals and perspiration for lactate measurements. The design must also minimize sensor-body motion, must minimize co-interference between the sensing modalities and be low-cost. These requirements motivate a flexible epidermal electronic design that can be worn on the chest and fabricated using screen-printing technology.

**Figure 1:**
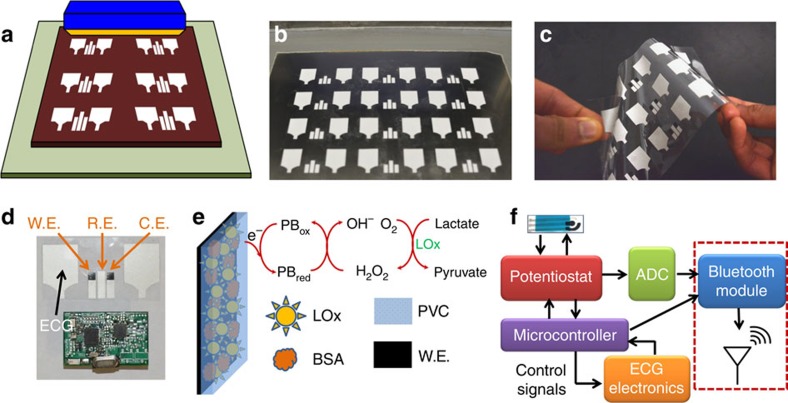
Fabrication and function of the Chem–Phys hybrid sensor patch. (**a**) Schematic showing the screen-printing process. (**b**) Image of the Chem–Phys printing stencil. (**c**) An array of printed Chem–Phys flexible patches. (**d**) Image of a Chem–Phys patch along with the wireless electronics. (**e**) Schematic showing the LOx-based lactate biosensor along with the enzymatic and detection reactions. (**f**) Block diagram of the wireless readout circuit.

The design of the sensing system is shown in Fig. 1. The biosensors were fabricated via conventional low-cost screen-printing technique (conceptually illustrated in Fig. 1a) utilizing custom-designed stencils (photograph in Fig. 1b). The biosensing patches were printed onto a highly flexible, thin polyester sheet (50 μm thickness) for realizing highly conformable sensor patch that adheres well to the human skin without causing any discomfort. An array of fabricated sensors is shown in Fig. 1c. The total patch size was dictated by the bipolar electrocardiogram electrodes, which must be separated by a minimum distance to attain a high-quality signal^48^. Typically, single-lead monitoring systems, such as the present design, are used for basic heart monitoring, arrhythmias diagnosis or studying the effect of exercise on the heart, and are placed in the vicinity of the conventional V1–V6 chest-lead locations. Electrode size, separation and placement parameters were determined through a series of experiments involving the placement of Ag/AgCl-based electrocardiogram electrodes of various sizes (1 × 1, 1.5 × 1.5, 1.5 × 1 and 2 × 2 cm^2^) and separation distances (1–6 cm) on subjects with different chest sizes, and observing the resulting electrocardiogram waveforms. The study revealed that a compact patch that provides favourable electrocardiogram signal could be realized by placing 1.5 × 1.5 cm^2^ electrocardiogram electrodes across the V1 and V2 lead sites with an inter-electrode distance of 4 cm, thereby measuring from the vantage point of the septal surface of the heart as suitable for diagnostics of arrhythmias and the effects of exercise on the heart. This sets an upper-end size of the patch to be 7 × 2 cm^2^. The chest region is not only convenient for measurement of electrocardiogram, but also has a high-sweat rate during physical excursion^49^,^50^, and can thus serve as an appropriate location to also measure lactate levels in human perspiration. In addition, the epidermis and muscle tissues over these locations do not experience complex 3D strains and remain fairly stable even during intense physical activities, making measurements here especially convenient. Since the performance of amperometric lactate electrodes is not compromised by reducing their dimension, they were fabricated between the two electrocardiogram electrodes, as shown in Fig. 1d. Each of the three electrodes have an active area of 3 × 2.5 mm. The working electrodes were printed using prussian blue ink due to the high selectivity of prussian blue towards hydrogen peroxide, a byproduct of the enzymatic oxidation of lactate^38^,^51^ (Fig. 1e). The reference electrode was printed using Ag/AgCl. Since sweat can provide an alternate electrically conductive pathway between the electrocardiogram electrodes and also between the electrocardiogram and amperometric electrodes, thus leading to a potential distortion of the recorded electrocardiogram signal, a printed hydrophobic layer of Ecoflex was used to separate the amperometric biosensor from the electrocardiogram electrodes. This effectively increases the impedance between the electrocardiogram and amperometric electrodes via sweat, thus minimizing the cross-talk between the two sensors. The entire Chem–Phys patch is highly flexible and can be smoothly mated on curved surfaces. Such flexibility is crucial for achieving unobtrusive wearable devices that cause no hindrance or irritation to the wearer.

The Chem–Phys patch was interfaced to a custom-PCB featuring a potentiostat and analogue-to-digital converter for amperometric data acquisition, an AFE for electrocardiogram data acquisition, and a BLE chip for wireless transmission (Fig. 1d,f).

### *In-vitro* characterization of the lactate biosensor

**Figure 2:**
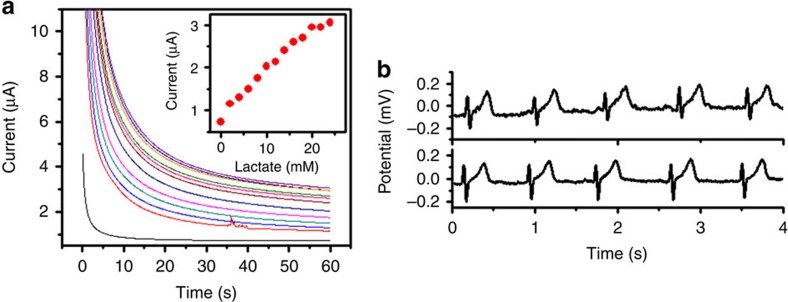
*In-vitro* characterization of Chem–Phys hybrid patch. (**a**) Amperometric response to increasing lactate concentration from 0 to 28 with 2 mM additions in phosphate buffer (pH 7.0). Applied voltage=−0.1 V versus Ag/AgCl. (**b**) Electrocardiogram signals using 3M Red Dot electrodes (top), and printed electrocardiogram sensor (bottom).

Lactate concentration in human sweat depends on a person's metabolism and level of exertion, and typically ranges from 0 to 25 mM (ref. 52). A wide linear-detection range, coupled with a fast response time is thus essential for continuous epidermal monitoring of lactate. The operating potential of −0.1 V (versus to Ag/AgCl) was selected based on the onset potential for electro-oxidation of lactate by the fabricated biosensor, obtained during cyclic voltammetry studies. When the biosensor comes in contact with lactate, the immobilized LOx enzyme catalyses the oxidation of lactate to generate pyruvate and H_2_O_2_. The prussian blue transducer then selectively reduces the H_2_O_2_ to generate electrons to quantify the lactate concentration (Fig. 1e). Figure 2a shows the amperometric response of the lactate biosensor to increasing lactate concentrations in the physiological range of 0–28 mM. It is evidenced from this figure that the biosensor responds linearly to the lactate concentrations in this range with a sensitivity of 96 nA/mM.

### On-body characterization of the electrocardiogram electrodes

The ability of the printed electrocardiogram electrodes to record electrocardiogram signals was validated by comparing recordings from the fabricated electrodes with commercially-available 3M Red Dot electrocardiogram electrodes. As illustrated in Fig. 2b, on-body electrocardiogram signals recorded for the same subject using commercial and fabricated electrodes at the same location have similar morphologies when acquired using the same AFE circuitry. All on-body experiments were performed in strict compliance with the guidelines of Institutional Review Boards (IRB) and were approved by the Human Research Protections Program at University of California, San Diego (Project name: Epidermal Electrochemical Sensors and Biosensors. Project number: 130,003).

### Epidermal evaluation of the Chem–Phys patch

**Figure 3:**
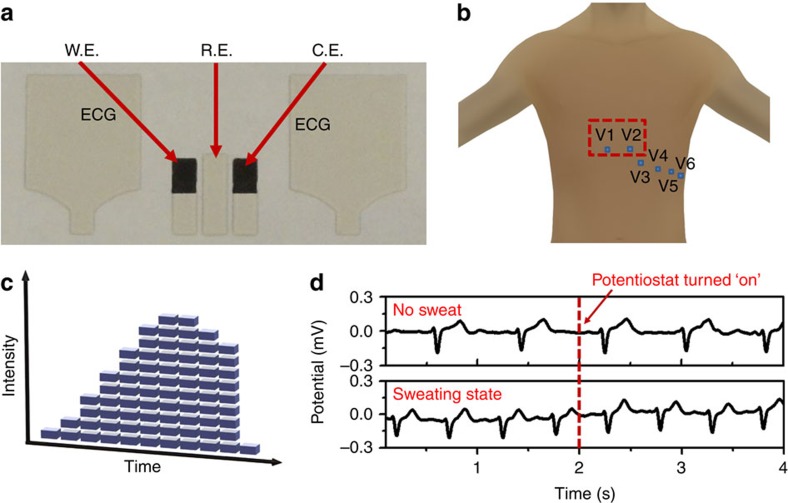
On-body test configuration. (**a**) A photograph of Chem–Phys hybrid patch. (**b**) Location of the Chem–Phys patch for mounting on the human body—the fourth intercostal space of the chest. (**c**) Cycling resistance profile for on-body tests. (**d**) Effect of amperometric measurement on the electrocardiogram signal before cycling (no sweat state) and during cycling (sweating state).

The Chem–Phys hybrid patch (Fig. 3a) was fabricated and applied to three healthy male subjects on the fourth intercostal space of the chest (Fig. 3b). Dynamic changes in sweat-lactate levels and electrocardiogram signals were measured continuously during a bout of intense cycling. To ensure that the anaerobic metabolism was invoked, subjects were asked to mount a stationary cycle and maintain a steady cycling cadence while the cycling resistance increased periodically as illustrated in Fig. 3c.

Since electrocardiogram measurements were made via bipolar high-impedance electrodes, and lactate measurements were made by applying a constant potential via a low-impedance potentiostat output and measuring current, there is a possibility that a change in the applied potentiostat voltage (for example, during start-up) could interfere with electrocardiogram measurements during the settling time of the potentiostat. At the same time, sweat consists of many ions and could thus act as an electrically conductive medium that can shunt the lactate and electrocardiogram sensors, or the two electrocardiogram electrodes together. Co-sensor interference and shunting effects were mitigated by geometrically separating the lactate and electrocardiogram electrodes and printing two vertically oriented hydrophobic layers were next to the lactate biosensor, thereby facilitating flux of new perspiration across the biosensor itself, while minimizing shunting between the lactate and electrocardiogram sensors. To validate performance under concurrent hybrid sensing scenarios, the Chem–Phys sensor was mounted on a human subject and set to continuously record electrocardiogram before, during, and immediately after turning on the −0.1 V potentiostat output. Experimental results, obtained via a wireless Bluetooth link as shown in Fig. 3d, reveal that the potentiostat has a negligible effect on the morphology of the electrocardiogram signals, irrespective of whether the subject was in a resting or cycling state.

**Figure 4:**
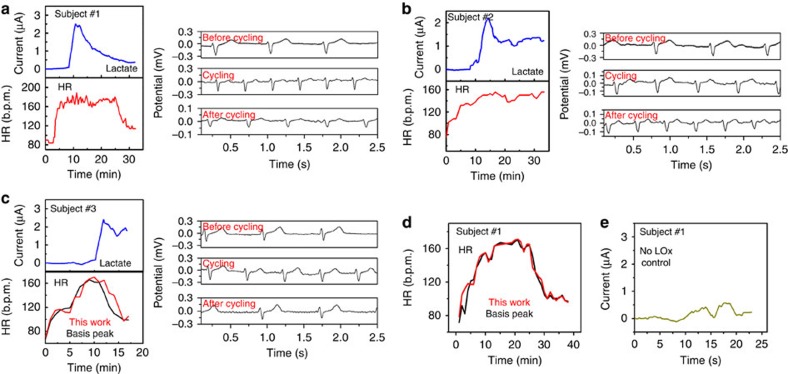
Real-time on-body evaluation of the Chem–Phys hybrid patch showing the lactate levels and heart rate for three human subjects. (**a**–**c**) The corresponding blue plots represent the real-time lactate concentration profiles for each subject, while, the red plots depict the heart rate data obtained by the electrocardiogram electrodes of the Chem–Phys patch. The black plots correspond to the heart rate data recorded by the Basis Peak heart rate monitor. Typical real-time electrocardiogram data obtained before, during and after the cycling bout for each subject is also shown. (**d**) Additional heart rate data from subject #1. (**e**) Response of the control amperometric sensor (without LOx enzyme) for subject #1.

To validate performance under realistic conditions, the Chem–Phys patch was tested on three subjects during 15–30 min of intense cycling activity; continuous time-series results during each experiment are shown in Fig. 4. At the commencement of the cycling activity, each subject's heart rate, extracted from electrocardiogram data, was within the normal resting range of 60 to 120 beats per minute (b.p.m.)^53^. At the same time, a negligible current response was measured by the lactate biosensor due to the lack of perspiration. With time, the resistance for cycling was increased, causing the subjects to exert increasing levels of effort to maintain constant cycling speed. This resulted in increasing heart rate and generation of sweat. At the onset of perspiration, lactate is released from the epidermis, and is selectively detected by the LOx-based biosensor. As the resistance increases, the sweat-lactate concentration too increases, as illustrated in Fig. 4a–c, showing a correlation between physical exertion, heart rate and, after a physiologic time delay, lactate generation. As the cycling continued, the sweat rate for each subject increased, leading to the well-documented phenomenon of dilution factor that causes decrease in the lactate concentration^42^. The final stage of the cycling bout involved a 3 min cool-down period. During this phase, as expected, the heart rate normalized back near to the normal resting heart rate. At the same time, the lactate concentration measured by the lactate biosensor continued to decrease.

The lactate biosensor data for each subject resembles the expected sweat-lactate profile for increasing intensity workouts^37^. To validate that lactate, not other sweat constituents, was specifically measured, a control experiment in which an unmodified (LOx-free) amperometric biosensor was used under the same experimental conditions as above to subject #1. As shown in Fig. 4e, the control biosensor leads to a negligible current response without the presence of LOx, confirming the high selectivity of the lactate biosensor. To validate electrocardiogram data over long time series, even under the presence of experimentally-induced motion, heart rate as extracted from the electrocardiogram data is benchmarked against a commercial wristband heat rate monitor (BASIS) for subjects 1 and 3. Extracted heart rate data matched the wrist-worn device with a Pearson's correlation coefficient of *r*=0.975. These on-body studies illustrate that the hybrid patch could monitor sweat lactate and electrocardiogram in a continuous and simultaneous manner, and that the hydrophobic barrier between the sensors assisted in minimizing potential cross-talk between the two sensing modalities. The data also demonstrates that such a barrier had minimal effect on the supply of oxygen to the enzyme electrode required for biocatalytic detection of lactate.

## Discussion

The Chem–Phys sensor patch described in this study represents a hybrid system that fuses the monitoring of electrophysiology with on-body chemical sensing into single fully printable wearable platform. On-body epidermal testing in a realistic fitness environment revealed that electrocardiogram sensing is in-line with existing wearable devices, and is not adversely affected by simultaneous measurement of lactate via constant-potential amperometry. The lactate control study using an enzyme-free amperometric sensor and correlation of the heart rate data of the hybrid patch to that recorded by a commercial heart rate monitor underscore the promise of the Chem–Phys patch to simultaneously monitor electrocardiogram signals and sweat-lactate levels for tracking the wearer's physicochemical and electrophysiological status. This device represents an important first step in the research and development of multimodal wearable sensors that fuse chemical, electrophysiological and physical sensors for more comprehensive monitoring of human physiology.

## Methods

### Reagents and materials

Chitosan, acetic acid, polyvinyl chloride, tetrahydrofuran, bovine serum albumin, L-lactic acid, sodium phosphate monobasic and sodium phosphate dibasic were obtained from Sigma-Aldrich (St Louis, MO). L-LOx (activity, 101 U mg^−1^) was procured from Toyobo Corp. (Osaka, Japan). All reagents were used without further purification. Prussian blue conductive carbon (C2070424P2), Ag/AgCl (E2414) and insulator (Dupont 5036) inks were procured from Gwent Group (Pontypool, UK), Ercon Inc. (Wareham, MA) and Dupont (Wilmington, DE). Electrocardiogram hydrogel conductive adhesive (RG63B, 35-mil thick) was purchased from Covidien. Polyester sheets (MELINEX 453, 50-μm thick) were provided by Tekra Inc. (New Berlin, WI).

### Instrumentation

The Chem–Phys patch was printed by using an MPM-SPM semiautomatic screen printer (Speedline Technologies, Franklin, MA). Sensor patterns were designed in AutoCAD (Autodesk, San Rafael, CA) and outsourced for fabrication on stainless steel through-hole 12 × 12 in framed stencils (Metal Etch Services, San Marcos, CA). Electrochemical characterization was performed at room temperature using a CH Instruments electrochemical analyser (model 630C, Austin, TX). A CONTEC MS400 Multi-parameter Patient Simulator, electrocardiogram simulator has been utilized for testing of electrocardiogram instrumentation circuits. 3M Red Dot multi-purpose monitoring electrodes are used for the verification of collected signal using the fabricated electrocardiogram sensors.

### Fabrication of Chem–Phys hybrid device

The Chem–Phys hybrid patch was fabricated via screen-printing technology, while the wearable electronic board was realized by relying on standard 4-layer PCB fabrication and assembly protocols.

### Printing and functionalization of Chem–Phys hybrid patch

The Chem–Phys patch was fabricated in-house by printing a sequence of Ag/AgCl, Prussian blue and insulator inks were patterned on the highly flexible transparent polyester substrate by using the custom-designed stencils and screen printer. The Ag/AgCl and insulator ink was cured at 90 °C for 10 min, while the Prussian blue ink was cured at 80 °C for 10 min in a convection oven.

On printing of the hybrid patch, the working electrode of the amperometric sensor was functionalized with LOx enzyme. The LOx solution (40 mg ml^−1^ containing 10 mg ml^−1^ bovine serum albumin stabilizer) was mixed with a chitosan solution (0.5 wt% in 1 M acetic acid) in a 1:1 v/v ratio. Subsequently, an 8-μl droplet of the above solution was casted on the electrode and dried under ambient conditions. Thereafter, 4 μl of polyvinyl chloride solution (3 wt% in tetrahydrofuran) was drop casted and allowed to dry under ambient conditions for at least 3 h before use. The electrocardiogram electrodes were covered with conductive hydrogel adhesives. The patch was then affixed to a medical-grade adhesive sheet required for applying to human skin. The patch was stored at 4 °C when not in use.

### PCB fabrication

The 4-layer Bluetooth-enabled PCB used a Texas Instrument (TI) CC2541 BLE System-on-Chip for communication and processing. An ADS1293 AFE chip was used for biopotential measurements to record the electrocardiogram (electrocardiogram) signals from the fabricated electrocardiogram electrodes. An LMP91000 AFE, programmable through an I2C interface driven by the CC2541, was used as the on-board potentiostat for lactate concentration determination. The data from each sensor was collected by the CC2541 and transmitted to a Bluetooth 4.0-enabled receiver. A graphical interface was developed using Python to demonstrate measurement results on a PC. A Johanson Technology 2.45 GHz chip antenna (2450AT42A100) and impedance-matched balun (2450BM15A0002) were used for wireless transmission. A CR2032 button cell lithium battery (3 V, 220 mAh) was utilized as a power source, regulated for the electronics via a TPS61220 boost converter. In the ‘active mode', the board consumed, on average, 5 mA from a 3 V supply (15 mW).

### *In-vitro* studies

*Characterization of amperometric lactate sensor*. These studies were performed using a 0.1 M phosphate-buffered solution (pH 7.0). The operating potential for the lactate sensor was selected by using cyclic voltammetry. The amperometric response was recorded after 1 min incubation in the sample solution, using a potential step to −0.1 V (versus Ag/AgCl) for 60 s.

*Characterization of the electrocardiogram sensor*. Electrocardiogram monitoring has been performed using both commercial 3M Red Dot Multi-Purpose monitoring electrodes, as well as fabricated electrocardiogram electrodes to verify the functionality of the printed Ag/AgCl electrocardiogram sensors.

*On-body characterization of Chem–Phys patch*. All experiments were performed in strict compliance with the guidelines of IRB and were approved by Human Research Protections Program at University of California, San Diego (Project name: Epidermal Electrochemical Sensors and Biosensors. Project number: 130,003). The study was deemed by the IRB as posing ‘no greater than minimal risk' to the prescreened subjects who were recruited for the investigation. A total of 3 healthy male volunteers (recruited in response to follow-up from flyers) with no prior medical history of heart conditions, diabetes or chronic skeletomuscular pain were recruited for participation in the study, and informed, signed consent was obtained from each individual following a rigorous prescreening procedure. A typical study comprised of applying the Chem–Phys hybrid patch on fourth intercostal space of a subject's chest to record the electrocardiogram signal between V1 and V2 positions.

Subjects were then asked to mount a stationary cycle and begin cycling at a steady, comfortable cadence. Subjects were instructed to maintain their cadence while an increasing resistance was applied at 3 min intervals. The absolute resistance level and duration was selected according to subject's fitness level while the same intensity profile was used throughout the human studies. This ensured that the anaerobic metabolism was invoked at similar time scales, hence augmenting the excretion of lactate in the perspiration in a controlled manner. Following the intense fitness bout, the volunteers were asked to gradually reduce their cadence during a 3 min ‘cool-down' period whereby the resistance was reduced from maximal levels.

*Characterization of instrumentation circuits*. The printed circuit board was assembled and tested *in-vitro* to validate both functionality and performance. The potentiostat circuit was verified together with the lactate biosensor through an *in-vitro* amperometric experiment. The electrocardiogram AFE was characterized using a CONTEC MS400 Multi-parameter Patient Simulator (electrocardiogram simulator). The output signal of the electrocardiogram simulator was read using ADS1293 AFE chip, and transferred through BLE link to a BLE-enabled device.

### Data availability

All relevant data are available from the authors on request.

## Additional information

**How to cite this article:** Imani, S. *et al*. A wearable chemical–electrophysiological hybrid biosensing system for real-time health and fitness monitoring. *Nat. Commun.* 7:11650 doi: 10.1038/ncomms11650 (2016).
